# Correction: Nuclear receptor LRH-1/NR5A2 is required and targetable for liver endoplasmic reticulum stress resolution

**DOI:** 10.7554/eLife.10084

**Published:** 2015-07-24

**Authors:** Jennifer L Mamrosh, Jae Man Lee, Martin Wagner, Peter J Stambrook, Richard J Whitby, Richard N Sifers, San-Pin Wu, Ming-Jer Tsai, Francesco J DeMayo, David D Moore

Mamrosh JL, Lee JM, Wagner M, Stambrook PJ, Whitby RJ, Sifers RN, Wu SP, Tsai MJ, DeMayo FJ, Moore DD. 2014. Nuclear receptor LRH-1/NR5A2 is required and targetable for liver endoplasmic reticulum stress resolution. *eLife* **3**:e01694. doi: 10.7554/eLife.01694

Published 15 April 2014

During quality control of our microarray dataset GSE68718 for inclusion in the NURSA Transcriptomine database (Ochsner et al. Physiol. Genomics 44, 853–863), we noticed a significant effect of the vehicle for DLPC (a 4:1 mixture of PEG-400 and Tween-80 delivered to mice by oral gavage twice daily) on gene expression. Genes most affected were generally those already induced strongly by tunicamycin. We did not detect this vehicle effect in previous experiments not utilizing ER stressors, indicating that this vehicle may behave unexpectedly in the context of ER stress. Unfortunately, this confounds the normalization and statistical assessment of the vehicle and vehicle + DLPC components of the dataset and we have therefore removed these data from Table 1 in our manuscript and from GSE68718. We did not observe similar effects of the vehicle (DMSO) for the LRH-1 agonist RJW100 on ER stress-induced gene expression in primary hepatocytes. Therefore, this correction does not conflict with our previous conclusions that LRH-1 agonism results in the induction of LRH-1 and ATF-2 target genes and promotes cell survival during ER stress. An outside analysis of the main microarray data presented in this paper (Table 1 excluding DLPC data and Figure 4) found the conclusions drawn to be accurate.

This article has been corrected accordingly. We apologize for the mistake.

