# Supplementary material for: Nuclear receptor LRH-1/NR5A2 is required and targetable for liver endoplasmic reticulum stress resolution
Source: eLife. 2014 Apr 15;3:e01694. doi: 10.7554/eLife.01694 (PMC3987120; doi:10.7554/eLife.01694)
Supplement: Supplementary file 1. — Primer sequences. DOI: http://dx.doi.org/10.7554/eLife.01694.014 [file elife01694s001.docx]

| Primers for qPCR: | | |
| --- | --- | --- |
| **Name:** | **Forward primer (5’ to 3’):** | **Reverse primer (5’ to 3’):** |
| *Arfgap3* | tttggaattcaagcccagac | aaccgtagcgatcctgaatg |
| *Atf2* | ctccagctcacacaactcca | tgtttcagctgtgccacttc |
| *Atf3* | gagatgtcagtcaccaagtc | cagtttctctgactctttctgc |
| *Atf6* | gcccagactgttttgctctc | cccatacttctggtggcact |
| *B3gat3* | ccagttgcaagctgatctcc | ctctcagcgtcctccactag |
| *Chop* | cggaacctgaggagagagtg | cgtttcctggggatgagata |
| *Creld1* | tgttccgattccctgaagct | aaacacgccgaacataccag |
| *Cyp7A1* | caagaacctgtacatgaggga | cacttcttcagaggctgcttt |
| *Cyp8B1* | gccttcaagtatgatcggttcct | gatcttcttgcccgacttgtaga |
| *Derl1* | gggggagtttgaaaaagagc | gctcaagtggcagtcctttc |
| *Derlin-3* | atgctggaggagggttcttt | agtgctgtcagagtgggctt |
| *Edem1* | tgggctggattccttctatg | ggtgggtctccttctccttc |
| *Ero1B* | tgattcgcaggaccacttttg | tagccagtgtaccgttccgg |
| *Ero1L* | gactgtgttggctgcttcaa | ccgtcctcctcagtgaacat |
| *Grp94* | ctgggtcaagcagaaaggag | tctctgttgcttcccgactt |
| *Ire1a* | cccaaatgtgatccgctact | ttgagagaatgcaggtgtgc |
| *Lrh-1* | ccctgctggactacacggttt | gctaatgggagatgtgacaaa |
| *Mcfd2* | gtttgcttgacggcctagag | accagacacgtccatcacat |
| *Plk3* | acctacagcaccgccatatc | cgcaggtagtagcgaacctc |
| *Sdf2l1* | gcggccaacagtcggtaa | gcgaatccgccagtaactattg |
| *Shp* | aacctgccgtccttctgcca | tatactgggcaccggagcct |
| *Ssr3* | ttgactgtttcctgcgactg | actctgcctgccacagattt |
| *Tbp* | gaagctgcggtacaattccag | ccccttgtacccttcaccaat |
| *Tmed3* | tattgccctgtttgtggtca | tctgtgagcttccgttgttg |
| *Tmed9* | acctccactcctggaccttt | cctgctcaaaatcccacatt |
| *Xbp-1 unspliced (Xbp-1u) ** | aagaacacgcttgggaatgg | actccccttggcctccac |
| *Xbp-1 spliced (Xbp-1s) ** | gagtccgcagcaggtg | gtgtcagagtccatggga |
|  |  |  |
| Primers for Chromatin Immunoprecipitation (ChIP)-qPCR: | | |
| **Name:** | **Forward primer (5’ to 3’):** | **Reverse primer (5’ to 3’):** |
| *B3gat3 CRE* | actcagtgacaagggtggag | tcccttactagttcccggga |
| *Creld1 CRE* | ggcgttgaagaagggtgtac | gggccaaaattagagagaggg |
| *Mcfd2 CRE* | gggacaggagttaggggttt | atccacgtgtcccctgtg |
| *Plk3 LRH-1 binding site* | cccggtgtttcgtcatctc | gaaagaggcctccggataac |

* from the following:

Zhang K, Kaufman RJ. 2008. Identification and characterization of endoplasmic reticulum stress-induced apoptosis i*n vivo*. *Methods Enzymol* **442**: 395-419.
